# The role of neighbourhood greenspace in children's spatial working memory

**DOI:** 10.1111/bjep.12243

**Published:** 2018-09-05

**Authors:** Eirini Flouri, Efstathios Papachristou, Emily Midouhas

**Affiliations:** ^1^ Department of Psychology and Human Development UCL Institute of Education University College London UK

**Keywords:** children, greenspace, neighbourhood, neighbourhood deprivation, spatial working memory

## Abstract

**Background:**

Exposure to nature may be particularly beneficial for the brain regions that support spatial working memory, a strong correlate of academic achievement.

**Aims:**

To explore whether children living in greener neighbourhoods (wards) have better spatial working memory.

**Sample:**

Drawn from the UK's Millennium Cohort Study, the sample was 4,758 11‐year‐olds living in urban areas in England.

**Methods:**

We fitted two‐level regression models, with children nested in wards, before and after adjustment for confounders, including poverty, parental education, sports participation, neighbourhood deprivation, and neighbourhood history. Spatial working memory was measured using the Cambridge Neuropsychological Test Automated Battery Spatial Working Memory task. Greenspace was measured as the percentage of greenery in the child's ward.

**Results:**

Even after controlling for confounders, lower quantity of neighbourhood greenspace was related to poorer spatial working memory. Importantly, neighbourhood deprivation did not modify this relationship. Therefore, lower quantity of greenspace was related to poorer spatial working memory similarly in deprived and non‐deprived neighbourhoods.

**Conclusions:**

Children living in greener urban neighbourhoods have better spatial working memory. If this association is causal, then our findings can be used to inform policy decisions about both education and urban planning.

## Background

Neighbourhoods with greenspace, often protected by planning policy, are thought to make their adult residents healthier, fitter and slimmer (Ward Thompson & Aspinall, 2011). However, there has been relatively little research into the role of greenspace for children, especially for outcomes in the cognitive domain (Dadvand *et al*., 2015; Faber Taylor & Kuo, 2009, 2011; Faber Taylor, Kuo, & Sullivan, 2002; de Keijzer, Gascon, Nieuwenhuijsen, & Dadvand, 2016; Kuo & Faber Taylor, 2004; Mårtensson *et al*., 2009; Schutte, Torquati, & Beattie, 2017; Wells, 2000). This is unfortunate because there are certainly many reasons why exposure to greenspace would have cognitive benefits for them. First, greenspace provides opportunities for physical activity (Bell *et al*., 2008), associated with improved cognitive outcomes in children (Wells, 2000). Second, it is associated with air quality, in turn related to physical health (Schwartz, 2004), a correlate of cognition in children (Lande, Kaczorowski, Auinger, Schwartz, & Weitzman, 2003; Li, Dai, Jackson, & Zhang, 2008). Third, it can impact on children via their parents, for example, by promoting adult mental and physical health (White, Alcock, Wheeler, & Depledge, 2013), related, in turn, to child cognitive outcomes through parenting (Goodman & Gotlib, 1999). Finally, and more directly, exposure to natural, green settings restores attentional resources by imposing fewer demands on visual or auditory processing. Prolonged periods requiring the use of ‘directed’ attention result in mental fatigue, which is characterized by feeling irritable and being easily distractible. According to Attention Restoration Theory (ART), contact with nature can assist in recovery from the fatigue of directed attention (Kaplan, 2001) and increase self‐regulation (Faber Taylor *et al*., 2002) because natural settings and views, which appear to draw primarily on ‘involuntary’ attention, give directed attention a chance to rest. A recent review (Ohly *et al*., 2016) pointed out that links between exposure to natural environments and attention have been found in a small number of studies with small samples, providing some support for ART.

Spatial working memory (henceforth SWM) is one aspect of cognition that has, to our knowledge, not been linked to exposure to greenspace among children. Working memory represents a limited‐capacity store for maintaining information over a short‐term period as well as carrying out mental operations on the contents of this store (Baddeley, 1986). SWM, considered to be one of the four components of working memory (Ang & Lee, 2008; Jones, Farrand, Stuart, & Morris, 1995), refers to the retention and processing of visuospatial information and is strongly inter‐related with attentional control (Awh, Vogel, & Oh, 2006). In order to temporarily store and manipulate visuospatial material, as well as navigate and find objects, one must not only retain information on locations but also hold and manipulate information for short periods of time while concurrently inhibiting distracting information, an attentionally demanding task.

Although the relationship between greenspace and *spatial* working memory in children is yet to be explored, a gap we aimed to fill with this study, a recent study investigated the association between outdoor surrounding greenness at home and school and primary school children's attention and working memory (Dadvand *et al*., 2015) and found that greenspace, particularly around school, was positively linked with both cognitive outcomes. We expected that SWM would be strongly linked to the amount of area greenspace given that those living in areas with more greenspace are more likely to actively use outdoor spaces. Active exploration of an environment leads to better spatial learning and wayfinding than does passive exposure (Chrastil & Warren, 2012). Spatial learning and wayfinding, the ability to learn, remember and follow a route through the environment (Blades, 1991), are, in turn, strongly related to SWM (Fenner, Heathcote, & Jerrams‐Smith, 2000). ART would also predict a link between greenspace and SWM, albeit via a different mechanism (Kaplan & Berman, 2010) as explained above. According to ART, green spaces and natural environments, such as parks and gardens, engage involuntary attention while keeping the requirements to engage directed attention at a minimum. This, in turn, allows directed attention to be replenished, leading to better performance on tasks that depend on it.

We carried out this study to fill this gap in the literature and add to the evidence on the role of exposure to greenspace in child cognition in general. Using data from the UK's Millennium Cohort Study (MCS), a large population‐based longitudinal birth cohort, we explored the potential of quantity of greenspace in urban areas [i.e., settlements with populations of over 10,000 (Bibby & Shepherd, 2004)] in England to predict SWM in children aged 11 years (the first, and so far only, time SWM was measured in MCS). We explored this association in an urban sample, excluding rural dwellers, because neighbourhood greenspace may be confounded with levels of rurality (Mitchell & Popham, 2007; White *et al*., 2013). At the age 11 follow‐up, the majority of MCS participants (76.9%) lived in urban areas, indicating that the MCS population is heavily urban to begin with. We also investigated whether the association between urban greenspace and SWM differs by neighbourhood deprivation, which we took to approximate the ‘quality’ of the urban area and therefore, to an extent, the quality of the urban area's greenspace and its use. Research with adults suggests that the quality of greenspace may matter more than its quantity (Fuller, Irvine, Devine‐Wright, Warren, & Gaston, 2007). For example, in the United Kingdom, Mitchell and Popham (2007) reported poorer adult self‐rated health with increasing percentage of greenspace in suburban low‐income areas but not in more central urban or rural low‐income areas. They suggested that this may be due to poorer‐quality greenspace in low‐income suburban areas.

## Method

### Participants

**Figure 1:**
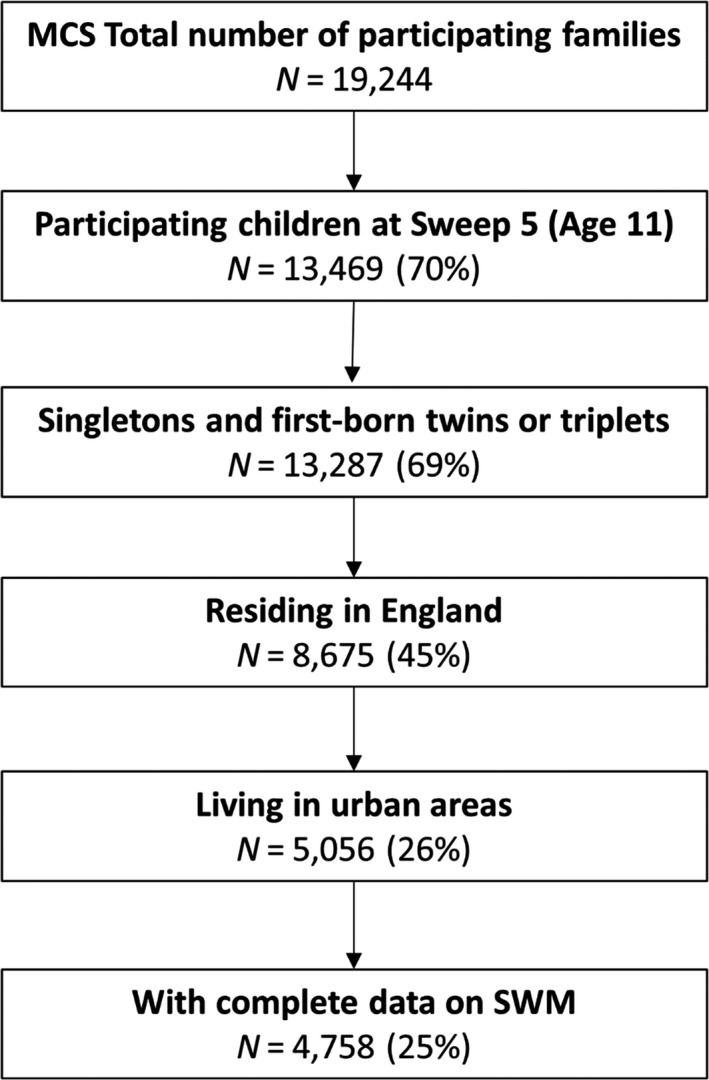
Flow chart of the analytic sample.

Our sample was drawn from the MCS, a cohort study of children born between 1/9/2000 and 31/8/2001 (England & Wales), or between 23/11/2000 and 11/01/2002 (Scotland and Northern Ireland) (Joshi & Fitzsimons, 2016). MCS children were followed from around 9 months to around 3, 5, 7, 11, and 14 years. MCS was designed to over‐represent families living in wards of high child poverty, wards with high proportions of ethnic minority populations across England, and the three smaller UK countries. (Wards are the key building blocks of UK administrative geography. They are the spatial units used to elect local government councillors. Population counts can vary substantially, but the average is about 5,500.) Parent‐reported data were collected through interviews and self‐completion questionnaires. Ethical approval was gained from NHS Multi‐Centre Ethics Committees, and parents gave informed consent before interviews took place, as did the cohort children themselves from age 11. MCS has data on SWM at age 11, when 13,287 families took part in the study. Of those, 5,056 lived in urban areas in England and all had data on neighbourhood greenspace at age 11. The study's analytic sample was children (singletons and first‐born twins or triplets) in urban England with data on SWM at age 11 (*N *=* *4,758, of whom 2,421 were male), clustered in 239 wards, with an average of 16.8 children in each (the number of children within wards ranged from 1 to 92). The selection of our analytic sample is outlined in the flow chart in Figure 1.

### Measures

#### Neighbourhood greenspace

**Figure 2:**
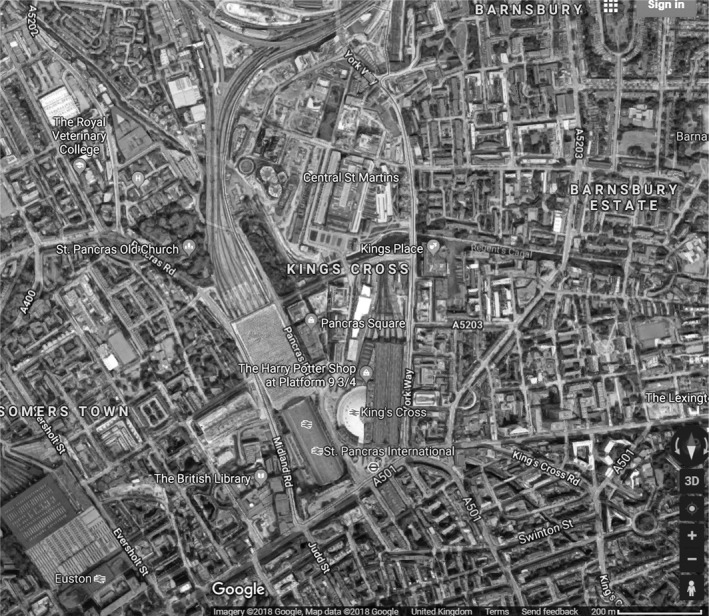
Satellite image of King's Cross (0% greenspace), Google June 2018. This is not the actual image used to retrieve ward‐level greenspace information and is to be used for illustrative purposes only.

**Figure 3:**
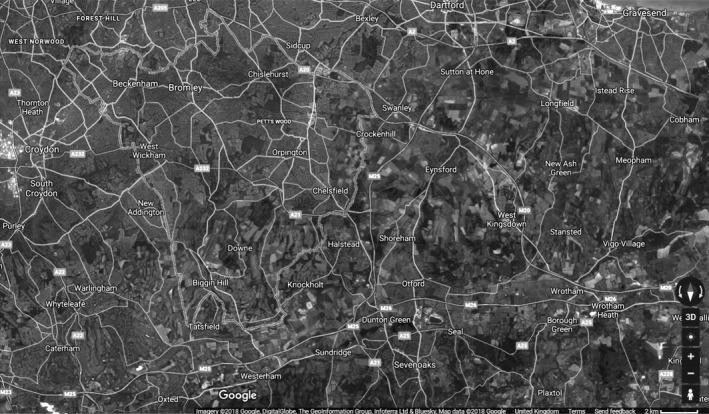
Satellite image of Bromley (90.5% greenspace), Google June 2018. This is not the actual image used to retrieve ward‐level greenspace information and is to be used for illustrative purposes only.

Neighbourhood greenspace at age 11 was measured with data from the Multiple Environmental Deprivation Index (MEDIx; http://cresh.org.uk/cresh-themes/environmental-deprivation/medix-and-medclass/), an ordered measure of physical environmental deprivation that represents the balance of pathogenic and salutogenic characteristics in a ward (Richardson, Mitchell, Shortt, Pearce, & Dawson, 2010). Greenspace, one of the components of MEDIx, across the United Kingdom at this level of geography was measured by combining land use data from the Coordination of Information on the Environment (CORINE; EEA, 2000) and the 2001 Generalised Land Use Database (GLUD; Office of the Deputy Prime Minister, 2005). CORINE is a land cover dataset from 2000 for the whole of the United Kingdom, derived from remotely sensed satellite imagery. It does not capture smaller green spaces (the smallest area mapped in the United Kingdom was roughly 1 ha) and therefore is only sensitive to larger green spaces such as parks. GLUD classifies land use at high geographical resolution (across England only) into nine categories (greenspace, domestic gardens, fresh water, domestic buildings, non‐domestic buildings, roads, paths, railways, and other) and offers an indicator of the percentage of greenspace per ward in England. GLUD estimates include all vegetated areas larger than 5 m^2^ in an area (with the exception of domestic gardens), regardless of their accessibility (public or private). CORINE and GLUD were used together to produce a dataset estimating greenspace within all wards in the United Kingdom (Richardson & Mitchell, 2010). In MCS, the percentages of ward‐level greenspace using this measure have been converted to deciles. In urban areas in MCS, the lowest decile corresponds to wards with less than 19% greenspace and the top to wards with 80 – 95%. (Across both rural and urban areas, the percentages are, respectively, <21% and >94%.) Urban areas in the United Kingdom therefore can vary substantially in amount of greenspace. The example images in Figures 2, 3 of two areas in London illustrate this clearly.

#### Spatial working memory

Spatial working memory at age 11 was measured with the Cambridge Neuropsychological Test Automated Battery (CANTAB) SWM task (Robbins *et al*., 1994) through a computer‐assisted personal interview. The task measures the ability to retain visuospatial information and to manipulate remembered items in working memory, and is one of the many available measures of SWM. Some of these measures focus more on visual memory (e.g., the Visual Patterns Test) and others more on spatial memory (e.g., the Corsi blocks task). Visual memory is responsible for retaining visual shapes and colours, whereas spatial memory for attending to locations and movement. These two forms of memory work together however. For example, to memorize a moving object's shape, one must utilize information about the space and where it is located (Klauer & Zhao, 2004). The CANTAB SWM task works on both forms of memory. The test begins with a number of coloured squares (boxes) being shown on the screen. Participants must search for blue tokens by touching the coloured boxes to open them. The task becomes more difficult as the number of boxes increases. The critical instruction is that the participant must not return to a box where a token has previously been found. The aim of the task is that, by touching the boxes and using a process of elimination, the participant should find one blue token in each of a number of boxes. The number of boxes is gradually increased, until it is necessary to search a total of eight boxes. The colour and position of the boxes used are changed from trial to trial to discourage the use of stereotyped search strategies. In our study, the outcome measure used was the number of ‘total errors’, that is, the number of times a participant touches a box that is certain not to contain a token. It is therefore the sum of errors made within searches (‘within errors’, i.e., whether the participant revisited a box known to be empty) and between searches (‘between errors’, i.e., whether the participant revisited a box where a blue token had already been found). A high number of total errors therefore indicates poor SWM.

#### Covariates

**Figure 4:**
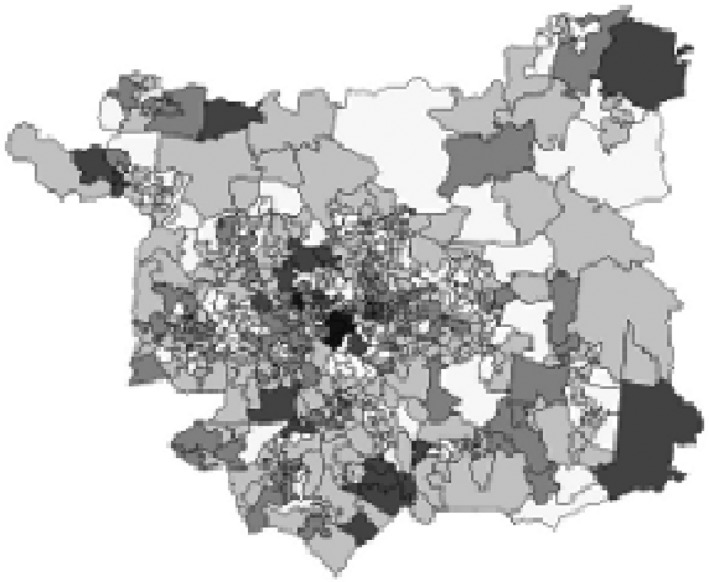
Map of LSOA boundaries in Leeds. Retrieved from https://census.ukdataservice.ac.uk/use-data/guides/boundary-data, 12th June 2018. This does not represent areas where MCS participants lived and is to be used for illustrative purposes only. Copyright statement: Contains National Statistics data © Crown copyright and database right 2012. Contains Ordnance Survey data © Crown copyright and database right 2012.

We controlled for confounding by adjusting for variables related to both families’ selection into neighbourhoods and children's SWM. These covariates were family socio‐economic status (Hackman *et al*., 2014; Maas, Verheij, Groenewegen, de Vries, & Spreeuwenberg, 2006), which we approximated by family poverty and maternal education, ethnicity (Archer & Francis, 2006; Comber, Brunsdon, & Green, 2008), sports participation (Brodersen, Steptoe, Boniface, & Wardle, 2007; McMorris, Sproule, Turner, & Hale, 2011), and computer gaming (Pujol *et al*., 2016; Veitch *et al*., 2011). Moreover, although we estimated cross‐sectional associations of neighbourhood greenspace and SWM at age 11, we controlled for residential mobility since infancy (as a proxy for neighbourhood history) to partial out any effects of different previous exposures to greenspace. Finally, to ensure that neighbourhood greenspace ‘effects’ are unconfounded by neighbourhood deprivation, we controlled for concurrent neighbourhood deprivation. We also adjusted for gender and exact age in months. All variables were measured at age 11 unless otherwise specified, as follows. Sports participation was based on the variable ‘How often do you play sports or active games inside or outside?’ Responses ranged from ‘0 = not at all/less than 1 day a week’ to ‘5 = 5 or more days a week’. Computer gaming was measured by a variable indicating whether the child played games on a computer or a console most days of the week or not. Maternal education was measured by a dummy variable indicating if the mother had a university degree or not. Poverty was measured as the average number of MCS sweeps (since the beginning of MCS at 9 months) during which the family's income was below the poverty line. Residential stability was measured with a dummy variable of living at the same address since the beginning of MCS or not. Neighbourhood deprivation was measured (in deciles, ranging from ‘1 = most deprived’ to ‘10 = least deprived’) by the level of deprivation using the Index of Multiple Deprivation (IMD) of the child's immediate area (Lower layer Super Output Area [LSOA]). LSOAs, typically smaller spatial units than wards, cover around 1,500 inhabitants, with boundaries drawn to maximize social homogeneity. Figure 4 shows an example of LSOA boundaries in Leeds to demonstrate the physical size of LSOAs. The 2004 IMD for England (the version of IMD that is linked to MCS and we therefore used) is based on data from the 2001 UK Census. It is a weighted aggregation of seven domains of deprivation measured at LSOA level. The domains are as follows: income deprivation (weighted at 22.5% of overall deprivation); employment deprivation (weighted at 22.5%); health deprivation and disability (weighted at 13.5%); education, skills and training deprivation (weighted at 13.5%); barriers to housing and services (weighted at 9.3%); living environment deprivation (weighted at 9.3%), and crime (weighted at 9.3%). For each domain, each LSOA in England is ranked in terms of how deprived it is relative to the other English LSOAS. IMD scores were constructed in two stages as follows. First, each domain rank was transformed to an exponential distribution. Then, the domains were combined using the domain weights shown above. Therefore, the IMD score for an LSOA is the combined sum of the weighted, exponentially transformed domain rank of the domain score (Noble *et al*., 2004 for further information).

### Analytic strategy

We fitted multilevel linear models (Snijders & Bosker, 1999) whereby child SWM was specified to vary randomly by sampling ward. As explained, MCS families were sampled from wards. Children living in the same ward are likely to share family and individual characteristics related to selective area sorting (Hedman & van Ham, 2012). Children's outcomes, including SWM, associated with these characteristics may therefore be correlated within wards. If this is ignored, then standard errors will be underestimated, increasing the probability of finding a statistically significant effect where one does not exist.

We started with the ‘null’ two‐level variance components model, in which the variation in SWM was modelled by a random intercept term for ward (Level 2) and a random error term for child (Level 1). Next, we introduced in the fixed part of the model the main study variables of greenspace, neighbourhood deprivation and their interaction term, alongside the additional individual‐level variables (Level 1) of gender, White ethnicity, exact age, maternal education, family poverty, sports participation, computer gaming, and residential stability. For both models, we calculated the intra‐class correlation coefficient (ICC) to estimate the variance in SWM that is attributable to ward. In both models, we used a study‐specific weight at Level 1 to account for the probability of non‐response and attrition, and controlled for the MCS stratum to account for the disproportionate stratification of the MCS sample. Collinearity among the predictor variables was tested by inspecting the variance inflation factor (VIF) estimates (VIF estimates >4 are considered indicative of multicollinearity). The level of significance was set at .05. We used listwise deletion throughout, so participants with missing data on a variable were left out of the models. The total number of participants excluded was 752 (16% of the analytic sample). Models were run using the *xtmixed* command in Stata/SE 14.2.

## Results

### Descriptives and bivariate analyses (unweighted data)

As explained, 5,056 families lived in urban areas in England at the time of the age 11 MCS follow‐up, of whom 4,758 had data on SWM, comprising our analytic sample. As expected, the 298 families who did not have data on SWM, and were therefore excluded from our analyses, were poorer, less educated and more likely to be non‐White. The amount of missing data in the analytic sample was low, ranging from 0% to 5% on the study variables (0.02% for White ethnicity, 1.6% for computer gaming and 4.7% for sports participation; the remaining variables had no missing values). The only exception was in the residential stability variable (10% of cases had missing values), which, however, was expected given that in creating this variable we had to exclude the ‘new families’ who joined MCS at Sweep 2 (for more details see the MCS technical report on sampling, http://www.cls.ioe.ac.uk/page.aspx?&sitesectionid=878&sitesectiontitle=Technical+Reports).

Table 1 describes the analytic sample's characteristics by quantity of neighbourhood greenspace. As expected, children in neighbourhoods with more greenspace made fewer SWM errors and were more likely to be White, come from socio‐economically advantaged families and participate in sports. Also as expected, families in such areas had been more residentially mobile (reflecting the tendency of UK families to move to neighbourhoods with more greenspace after children are born).

**Table 1:** Variable distribution by neighbourhood greenspace in the analytic sample

	Total *N* = 4,758	Low neighbourhood greenspace (Lower 3 deciles) *N* = 2,584 (54%)	High neighbourhood greenspace (Upper 7 deciles) *N* = 2,174 (46%)	*p*‐Value
Continuous variables, *M* (*SD*)				
SWM (total errors)	34.97 (0.27)	36.21 (0.39)	33.51 (0.39)	<.001
Age	10.63 (0.01)	10.63 (0.01)	10.64 (0.01)	.59
(Low) neighbourhood deprivation	5.34 (0.04)	4.66 (0.05)	6.16 (0.06)	<.001
Poverty	0.28 (0.01)	0.33 (0.01)	0.22 (0.01)	<.001
Categorical variables, *n* (%)				
White	3,532 (74%)	1,587 (61%)	1,945 (89%)	<.001
(No/low) sports participation	2,311 (51%)	1,321 (54%)	990 (48%)	<.001
Female	2,337 (49%)	1,528 (49%)	1,079 (50%)	.42
Computer gaming	2,567 (55%)	1,390 (55%)	1,177 (55%)	.92
Maternal education (degree)	1,051 (22%)	600 (23%)	451 (21%)	.04
Residential stability	1,726 (41%)	959 (43%)	767 (38%)	<.001

*Note*

*p*‐Values of *t*‐tests for continuous variables and chi‐square tests for categorical variables.

### Multilevel models

In the ‘null’ model, the child‐level variance component (Level 1 variance) was 17.96 (*SE *= 0.20), and the variance due to clustering in wards was 3.73 (*SE *= 0.44). The resulting ICC, specifying the variation in the dependent variable (in this case, SWM) due to the variation between groups (in this case, wards), was .041. This suggests that 4.1% of the variance in SWM was attributable to ward. The results of the fully adjusted multilevel model are presented in Table 2. Overall, children residing in wards with more greenspace and those in less deprived areas had better SWM (both *p*‐values < .05). Specifically, an increase in one decile across the distribution of wards by greenspace was associated with a decrease in roughly three‐fourths of an SWM error (*b *=* *−0.79, *SE* = 0.38). A decrease in one decile across the distribution of LSOAs by deprivation was related to a decrease in around two‐thirds of an SWM error (*b *=* *−0.62, *SE* = 0.28). The interaction term between greenspace and neighbourhood deprivation, however, was not statistically significant (*p *=* *.17), suggesting that the effect of greenspace on SWM was similar across levels of neighbourhood deprivation. (A supplementary analysis showed that the interaction between neighbourhood greenspace and residential stability was not significant either, suggesting that current contextual greenery was significant for those with different exposures, too.) Many of the covariates had significant effects. For example, total errors were associated positively with poverty, and negatively with age, maternal education, and sports participation. As expected, the amount of variance explained due to clustering in wards (σ^2^μ* *= 3.36, *SE* = 0.42) and the resulting ICC (3.6%) were reduced in the fully adjusted model compared to the ‘null’ model. VIF values were very low, ranging, in the fully adjusted model before entering the interaction term, from 1.00 (age) to 1.52 (poverty), suggesting that the standard errors of the regression coefficients were reliable.

**Table 2:** Fully adjusted two‐level regression model (fixed and random effects) predicting SWM (total errors)

	Coeff. (*SE*)	95% CI
Fixed effects		
Constant	70.391 (7.997)***	[55.257, 86.606]
Neighbourhood greenspace	−0.793 (0.384)*	[−1.545, −0.041]
Female	−1.201 (0.619)	[−2.414, 0.012]
Age	−2.758 (0.719)***	[−4.168, −1.348]
White	−1.484 (1.094)	[−4.952, −1.702]
Sports participation	−1.249 (0.200)***	[−1.641, −0.856]
Computer gaming	1.074 (0.608)	[−0.118, 2.267]
Maternal education (degree)	−3.327 (0.829)***	[−4.952, −1.702]
Poverty	5.570 (1.264)***	[3.093, 8.047]
Residential stability	−0.822 (0.646)	[−2.089, 0.445]
(Low) neighbourhood deprivation	−0.618 (0.278)*	[−1.162, −0.074]
Neighbourhood greenspace * neighbourhood deprivation	0.083 (0.060)	[−0.035, 0.201]
Random effects		
Between‐ward variability (σ^2^μ)	3.363*** (0.419)	[2.633, 4.294]
Between‐child variability (σ^2^ε)	17.494*** (0.207)	[17.092, 17.906]

*Notes*

Model results adjusted for stratum (to control for the disproportionate stratification of the MCS sample).

*p < .05; ***p < .001.

As this was complete case analysis, we also attempted to estimate sample bias. As expected, those of the analytic sample excluded from the multilevel models due to missing data did not differ from those with complete data (*N *=* *4,006; 84%) in terms of gender (*p *=* *.14), residential stability (*p *=* *.78), maternal education (*p *=* *.93), or age (*p *=* *.81). However, they were more likely to be non‐White (*p *<* *.001), and they tended to be poorer (*p *=* *.001), play organized sports less frequently (*p *<* *.01), play computer games more frequently (*p *=* *.03), have worse SWM (*p *=* *.001), and live in more deprived areas (*p *<* *.001) and areas with less greenspace (*p *<* *.001).

## Discussion

We carried out this study to explore the role of urban neighbourhood greenspace in 11‐year‐old children's SWM in England. To our knowledge, this is the first study to examine the association in children between quantity of neighbourhood greenspace and this particular aspect of working memory. We expected that SWM capacity would be related positively to the amount of neighbourhood greenspace for two reasons. First, children in areas with more greenspace are more likely to explore their outdoor environment. Active exploration of an environment is, in turn, linked to wayfinding, a strong correlate of SWM. Second, according to ART (Kaplan & Berman, 2010), green spaces – such as parks and gardens – engage bottom‐up attention. At the same time, in such contexts the requirements to engage top‐down attention are minimized. This in turn allows top‐down attention abilities to be restored and replenished, leading to better performance on tasks that depend on them.

Although we did not have data to test these two specific pathways, we found, as expected, that children living in urban neighbourhoods with more greenspace had better SWM, as measured by the CANTAB SWM task. That effect was robust to adjustment for family poverty, parental education, sports participation and neighbourhood deprivation, all associated with neighbourhood greenspace and child cognitive skills in general. The size of the effect, however, was quite small. Specifically, an increase in one decile across the distribution of neighbourhoods by greenspace was associated with a decrease in roughly three‐fourths of an SWM error. Nevertheless, this is a substantively important finding as it suggests that exposure to greenspace may have specific cognitive benefits for children. Importantly, neither neighbourhood history nor neighbourhood deprivation modified the effect of greenspace on SWM. That is, neighbourhood greenspace was related to children's SWM similarly in deprived and non‐deprived neighbourhoods, and similarly in children with different neighbourhood histories. Together, these findings add to the existing evidence about the positive role of greenspace in cognitive functioning in children (Dadvand *et al*., 2015). Arguably, they also have important practical implications. SWM is an important cognitive ability. It is not associated only with navigation and wayfinding more broadly. Visual and spatial awareness and the ability to process spatial information are also strongly related with academic achievement in children (St Clair‐Thompson & Gathercole, 2006), and particularly mathematics performance (van de Weijer‐Bergsma, Kroesbergen, & Van Luit, 2015). For example, in a very recent study, Li and Geary (2017) showed that SWM was the only working memory component to uniquely predict mathematics achievement and gains in mathematics achievement over time in children, even after controlling for intelligence. If the association we established between greenspace and child SWM is causal, then our findings can be used to inform policy decisions about both education and urban planning. For example, a strong case could be made for outdoor learning (Becker, Lauterbach, Spengler, Dettweiler, & Mess, 2017) and for easy access to urban greenspace (Wolch, Byrne, & Newell, 2014).

Our study is not without weaknesses, however. Its main limitation is that it is cross‐sectional and correlational, and therefore cannot establish if children's SWM and exposure to greenspace are causally related. Second, we only explored links with SWM. Only research comparing effects on several measures of cognitive functioning in children can determine whether greenspace ‘benefits’ are general or specific to SWM. Third, we only had 1‐item child‐reported measures of computer gaming and sports participation, and, importantly, we could not know if the children played sports indoors or outdoors. Fourth, we had to use 2001 data to measure the greenspace and deprivation of the English urban neighbourhoods in which our sample lived around 10 years later. We did not therefore account for change in the characteristics of neighbourhoods over time, making, instead, an assumption that area characteristics were time‐invariant. There is little research on the extent of area change in the United Kingdom due to the limited availability of longitudinal data on areas and the lack of comparability of area boundaries and data over time (Lupton & Power, 2004). Nonetheless, emerging evidence suggests that area deprivation (Kontopantelis *et al*., 2018) and other area characteristics (Gambaro, Joshi, Lupton, Fenton, & Lennon, 2016) do not change substantially over 10 years in the United Kingdom, at least in recent history. Fifth, we could not take into account the quality of greenspace, which may be a stronger predictor of its use and benefits. Sixth, we could not know how and how much the children used green spaces. Seventh, we did not consider the greenspace available in adjacent areas. Finally, we did not consider the role of other contexts whose characteristics are associated with both urban neighbourhoods’ greenspace and children's cognitive skills, such as schools (Wu *et al*., 2014), likely the reason why our model could not fully explain the between‐area variation in child SWM in our sample.

Despite these limitations, our study suggests that, in urban areas in England, neighbourhood greenspace and child SWM are inter‐related. Future studies should test how the association between contextual greenspace and child SWM compares to associations between contextual greenspace and other aspects of child cognitive functioning. Furthermore, they should use geographical information systems to capture proximity to greenspace, which may be particularly important for access, especially in children. They should also capture the quality and function of greenspace and include information about its use. With these changes, future research will be able to determine with greater precision what cognitive benefits immersion in, access to, and use of area greenspace may confer on children.

## Conflicts of interest declaration

None.
